# Wheelchair Pressure Ulcer Prevention Using FBG Based Sensing Devices

**DOI:** 10.3390/s20010212

**Published:** 2019-12-30

**Authors:** Cátia Tavares, M. Fátima Domingues, Tiago Paixão, Nélia Alberto, Hugo Silva, Paulo Antunes

**Affiliations:** 1 Department of Physics & I3N, University of Aveiro, Campus Universitário de Santiago, 3810-193 Aveiro, Portugal; tiagopaixao@ua.pt (T.P.); pantunes@ua.pt (P.A.); 2 Instituto de Telecomunicações, Campus Universitário de Santiago, 3810-193 Aveiro, Portugal; nelia@ua.pt (N.A.); hsilva@lx.it.pt (H.S.); 3 Department of Bioengineering, Instituto Superior Técnico, University of Lisbon, 1049-001 Lisboa, Portugal; 4 PLUX—Wireless Biosignals, S.A, Avenida 5 de Outubro n. 70, 1050-059 Lisboa, Portugal

**Keywords:** fiber Bragg grating, pressure ulcers, wheelchair, pressure sensors, breathing rate

## Abstract

In this work, a fiber Bragg grating (FBG) based sensing system for wheelchair pressure ulcer prevention was developed. Six FBGs were strategically positioned in a wheelchair to monitor the more prominent bone areas, namely scapulas (right (SR) and left (SL)), ischiatic zone (right (IR) and left (IL)), and heels (right (HR) and left (HL)). The sensing architecture was tested by a female user during pressure relief exercises, to verify its effectiveness on pressure monitoring. The proposed system proves to be a compact and reliable solution for wheelchair pressure ulcer prevention, making it a suitable alternative to existing conventional electronic sensors, with the advantage of being immune to electromagnetic interferences and usable in humid environments. In addition to the pressure, the breathing rate was also monitored. By combining the proposed sensing architecture with a wheelchair user detection software, it is possible to create alerts for the user to know when a new position should be adopted, in order to relieve the pressure in a specific area, thus avoiding one of the biggest problems for such patients, pressure ulcers.

## 1. Introduction

Pressure ulcers are localized lesions of the skin or underlying tissues that occur due to the decrease of the blood circulation inflicted by the increased pressure in specific zones of the body [1]. These injuries are directly related to life quality loss, higher risk of death, and higher healthcare costs due to all the treatment needed. In 2004, a scientific study revealed that in the USA, the cost of each pressure ulcer treatment could be up to $70.000/year, and the estimated total cost per year was around $1.6 billion [2]. There are more recent financial studies, but their focus is mainly on the specific causes of ulceration [3,4].

All patients who are permanently or even temporarily immobilized, for example in post-surgery situations, present high risk of developing pressure ulcers [5]. Wheelchair users, who are permanently immobilized, sitting in the wheelchair for more than eight hours a day, are one of the most at risk groups [5].

**Figure 1:**
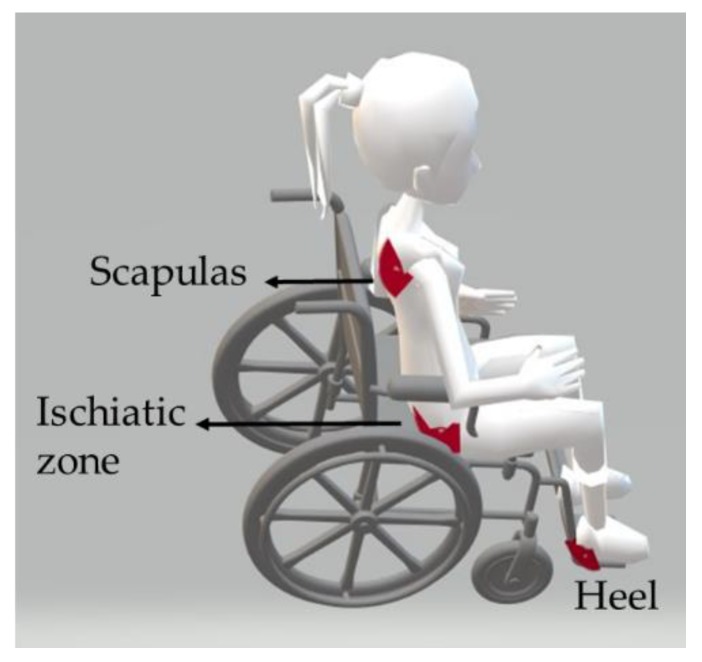
Representation of the most prominent bone zones for a wheelchair user.

For a wheelchair user, the prominent areas of the body where the user is seated (scapulas, ischiatic zones, and heels) are the most prone zones to develop this pathology, see Figure 1 [1].

Previous studies indicated that prevention is the key to reduce the health costs related to this pathology and improve the life quality of immobilized patients [2]. Thus, the scientific community has been investigating the influence of different parameters towards the reduction of the occurrence of this pathology, namely through the use of different support surfaces [6], types of diet [7,8], and patient repositioning [9,10]. At the same time, other scientific works have been published reporting the use of electronic sensors for wheelchair monitoring. These include devices for posture control and detection of the areas of higher pressure [1,5,10,11,12,13,14], sensors to identify fall risk postures [15], and a solution with an air cushion and pressure sensors that control the quantity of air in the cushion, according to the exerted pressure [16].

In this work, we present an alternative solution based on optical fiber Bragg grating (FBG) for the monitoring of pressure relief exercises for wheelchair users, targeting the prevention of ulcerations. Although the experimental setup used involves the use of an off-the-shelf interrogator, which may have some associated financial costs, this system can be replaced by other cost-effective interrogation techniques, like the ones based in edge filtering [17], which can considerably reduce both the size and the price of the overall sensing system.

Compared to their electronic counterparts, the advantages of the FBG technology include the ability to multiplex several sensors in one optical fiber, immunity to electromagnetic interferences, the possibility to be used in wet/humid areas without any encapsulation, and reduced weight and size [18,19,20,21]. Additionally, since no electrical current is needed at the measuring point, this sensing technique is intrinsically safer, allowing the design and implementation of a versatile and non-invasive sensing system, which can be used without affecting or compromising the user’s comfort. Note that this system will be in contact with the subject’s body, and the use of electronic sensors is not the most advisable, as they may suffer malfunctions due to the sweat of the body (humidity), or even be harmful to the patient/wheelchair user, due to the need to use electrical signals at the measuring point.

The proposed sensing system consists of a network with six FBGs sensors distributed over strategic zones of the wheelchair (chair back, chair seat, and footrest). The information retrieved with the proposed system will allow the monitoring of pressure relief exercises and further to evaluate if the users are performing them properly, and, consequently, to correctly instruct them. The potential connection of this sensing solution to an eHealth architecture [22] will also provide the medical staff with a continuous monitoring of the wheelchair user, offering a constant evaluation of the pressure exerted at the ulceration points. From the pressure results, the breathing rate can also be estimated. Based on the monitoring of such a vital sign, it is possible to detect critical situations as, for instance, the unconsciousness of the subject, or to collect relevant information for the early detection of diseases. This will contribute to increasing the quality and the efficiency of health treatment.

The paper is organized as follows. After this initial introduction (Section 1), Section 2 describes the produced FBG sensing element principle of operation. Section 3 focuses on the sensors development and their calibration results. The implemented experimental protocols, the results, and discussion are addressed in Section 4, and the conclusion is presented in Section 5.

## 2. Fiber Bragg Grating Sensing Principle

The pressure in different wheelchair zones was monitored using FBGs. These sensors are characterized by presenting a perturbation in the refractive index along the fiber core. When an optical fiber containing an FBG is illuminated by a broadband light source, only a set of wavelengths that meet the Bragg condition are reflected, all the others are transmitted. The Bragg condition is given by the following equation:

(1) $$\lambda_{B}=2n_{eff}\Lambda$$

where *λ_B_* is the reflected Bragg wavelength, *n_eff_* is the effective refractive index of the optical fiber core, and Λ is the grating period.

The *λ_B_* can be affected by changes in strain (Δl) and/or temperature (Δ*T*). Consequently, the reflected Bragg wavelength varies (Δ*λ_B_*), according to the following equation:

(2) $$\begin{array}{c} \Delta \lambda_{B}=\Delta \lambda_{B,\iota }+\Delta \lambda_{B,T}=2\left(\Lambda \frac{\partial n_{eff}}{\partial l}+n_{eff}\frac{\partial \Lambda }{\partial l}\right)\Delta l+2\left(\Lambda \frac{\partial n_{eff}}{\partial T}+n_{eff}\frac{\partial \Lambda }{\partial T}\right)\Delta T\\ \;=S_{l}\Delta l+S_{T}\Delta T \end{array}$$

where the first term is related to the strain induced wavelength shift, and the last to the thermal effect on the same parameter. *S_l_* and *S_T_* represent the strain and temperature sensitivity coefficients of the FBG.

**Figure 2:**
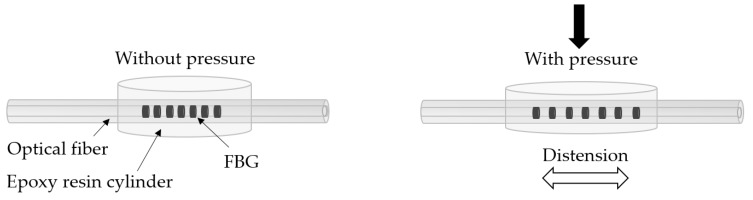
Sensor design and effect of pressure on the sensor.

Aiming to improve the implementation procedures and protect the physical integrity of the sensing elements when subjected to pressure variations, the FBGs for the pressure monitoring were embedded into a thermosetting epoxy resin (LiquidLens) cylinder (Figure 2). Thus, when a pressure is applied to the upper face of the resin cylinder, it will distend, causing a strain also in the optical fiber, and consequently modulating the reflected Bragg wavelength (Figure 2).

## 3. Sensing System Preparation and Calibration

In this work, eight FBGs were inscribed into photosensitive optical fiber (GF1 Thorlabs), using a pulsed Q-switched Nd: YAG laser system (LOTIS TII LS-2137U Laser), lasing at the fourth harmonic (266 nm). The FBGs were recorded through the phase mask technique, employing a laser pump energy of 25 J, a repetition rate of 10 Hz, and an exposure time of 1 min, approximately.

Six Bragg gratings were recorded and multiplexed in the same optical fiber cable, spaced so that it is possible to monitor the pressure at the interest points on the wheelchair, namely both scapulas (right (SR) and left (SL)), both ischiatic zones (right (IR) and left (IL)), and both heels (right (HR) and left (HL)). The other two FBGs were inscribed in separated optical fiber cables for the temperature monitoring.

Each of the six FBGs multiplexed in the same fiber cable were subsequently embedded in an epoxy resin cylinder of 2 cm diameter, with the sensors positioned in the middle point of the cylinder (called cell).

After the FBG embedding process, the cells were characterized to pressure variations, ranging from 0 to 319 kPa, using a Shimadzu^®^ AGS-5kND mechanical testing machine. Due to limitations in the equipment, a non-constant pressure step was used, however the range was always the same (from from 0 to 319 kPa). The tests were repeated three times for each cell, and additionally the hysteresis phenomenon was also evaluated by decreasing the pressure applied on the sensing cells after increasing. The response of the optical sensors was monitored using an interrogation system with an acquisition rate of 960 Hz (I-Mon USB 512, Ibsen).

**Figure 3:**
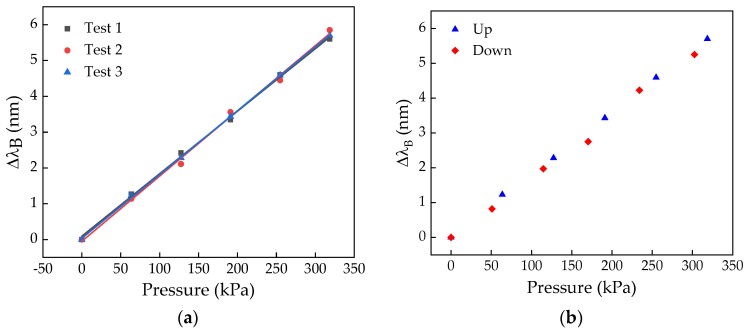
Results of the calibration (**a**) and hysteresis (**b**) tests for the left scapula (SL) cell.

The results of the calibration test for the SL cell, for the three tests, are shown in Figure 3a. In Figure 3b the experimental data obtained from the hysteresis test for the same cell is depicted.

For all the tests, a linear dependence of the Bragg wavelength shift with the applied pressure was verified, being the sensitivity coefficient given by the mean value of the slopes of the linear fits to the experimental data, in this particular case 17.8 ± 0.3 pm/kPa. As can be observed from the data in Figure 3b, the hysteresis effect is very low, and thus can be neglected.

Table 1 shows the sensitivity coefficient of each cell to the pressure variations. The values are not the same for all the cells, since it is difficult to precisely reproduce the sensing cells. However, this behavior has no influence on the final results, given that it is necessary to calibrate all cells individually, prior to their application.

Since the Bragg wavelength varies with pressure (induced strain) and temperature changes (Equation (2)), after the pressure calibration, and before the wheelchair pressure monitoring, the thermal sensitivity of the sensing cells was determined. The temperature was increased from 10.0 to 45.0 °C, with a step increment of 5.0 °C, using a climatic chamber (CH340, Angelantoni Industrie). For each temperature level, and after a stabilization period of 20 min, the reflected Bragg wavelength was registered. As for the pressure tests, different thermal sensitivity coefficients were obtained, ranging from 17.8 pm/°C to 19.1 pm/°C, for the SR and SL cells, respectively. These values are almost double with regard to the thermal sensitivity of 10 pm/°C obtained for a standard FBG. This difference is attributed to the thermal expansion coefficient of the epoxy resin where the FBGs were embedded [23].

## 4. Wheelchair Pressure Monitoring

### 4.1. Protocol and Implementation

**Figure 4:**
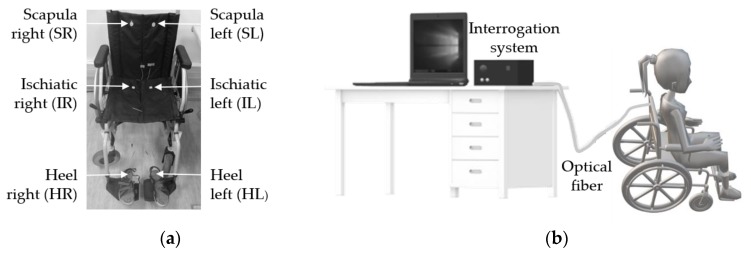
(**a**) Wheelchair with the six sensing cells; (**b**) Schematic representation of the experimental setup.

After the calibration process, the six cells were strategically placed in high pressure zones for wheelchair users, namely SL, SR, IL, IR, HL, and HR, as shown in Figure 4a. In the case of the scapulas and ischiatic zones, the cells were glued with a strong double-sided duct tape to the wheelchair, where it contacted with the bony prominence regions of the user. The sensors for heel pressure monitoring were implemented into cork insoles [22] of appropriate size, which were adapted to the user’s shoes. In Figure 4b we present a schematic representation of the overall experimental setup, which comprises the multiplexed FBG sensor network attached to the wheelchair and insoles, the optical interrogator, and the computer for data acquisition. The tests were realized in indoor conditions with the temperature almost constant during the entire experiment (21 °C).

With the cells placed in the target zones, a volunteer was asked to sit in the wheelchair and the FBG modulated signal was continuously monitored while the subject executed different pressure relief exercises. As the name suggest, the performed exercises were intended to relieve the pressure in areas most prone to pressure sores in wheelchair users, i.e., in scapulas, ischiatic, and heel zones [14].

**Figure 5:**
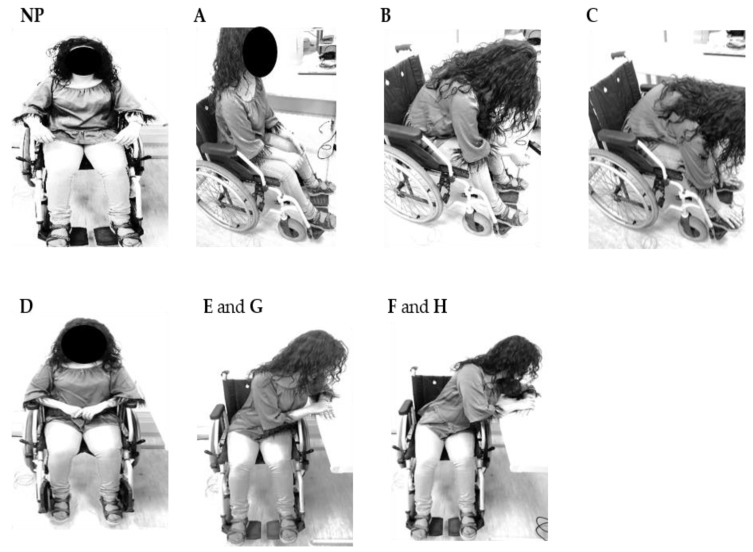
Representation of the nine positions adopted during the pressure relief tests.

Figure 5 shows the user positions during the exercises implemented for pressure relief, corresponding to the normal position (NP) and the different pressure relief situations: small frontward lean (A), intermediate frontward lean (B), full frontward lean (C), without feet support (D), intermediate sideward lean (Left—E; Right—G), and full sideward lean (Left—F; Right—H). To standardize the movements, the subject always put his hands, arms, and feet as represented in Figure 5. In the intermediate sideward lean position, only one scapula is in contact with the wheelchair, while in the full sideward lean position there is no contact with the wheelchair. In these positions (E, G and F, H), the arm is supported on a table, 10 and 20 cm apart from the wheelchair, respectively.

The tests were performed on a female person (37 years), without mobility pathologies, with the aim to assess the capability of this system to detect pressure variations during different pressure relief positions.

According to the work reported in [14], before performing any pressure relief position, the subject should be in the NP for at least 2 min, to stabilize blood flow, and therefore the pressure on the skin surface. Nevertheless, since the Bragg wavelength varies simultaneously with the pressure (strain) and the temperature (Equation (2)), a waiting period of 14 min (12 min plus the 2 min recommended on [14]) was used in the NP position, before the volunteer moved to the first pressure relief position (A). After this temperature stabilization period, a significant temperature variation was not expected to be registered during the tests. As suggested in the previous study [14], the volunteer was also asked to remain in the NP position for 2 min before moving to the other pressure relief positions. The relief positions were maintained for 1 min.

To investigate the thermal influence on the pressure values, two temperature sensors, consisting of FBGs inside a double needle [24] for strain isolation, were put on the wheelchair. One was positioned close to the right scapula, and the other close to the right ischiatic, since these were the areas in which a higher temperature variation was expected during the pressure relief exercises. The heels were not included in this study, since, except for the D position, the location of the feet remains unchanged. In the case of the D position, the feet are removed from the wheelchair supports, and although there is a pressure relief, it is not expected that there is a significant temperature change, since the feet remain in contact with the cells/cork insoles. This is corroborated by results of previous studies, regarding the use of FBG sensors in the production of an insole for gait analysis, which showed that the temperature variation for this type of application can be neglected [25].

The sequence of the pressure relief exercises carried out in this temperature test was the same as that described for the pressure test. Briefly, prior to any exercise, there was a stabilization period of 14 min (12 min + 2 min corresponding to the NP position), followed by several pressure relief positions during 1 min, intercalated by 2 min in the NP position. Note that in this work there is no temperature compensation on the pressure results, and the main aim of this test was to assess the error induced on the pressure results when the thermal effect is not compensated.

### 4.2. Results and Discussion

**Figure 6:**
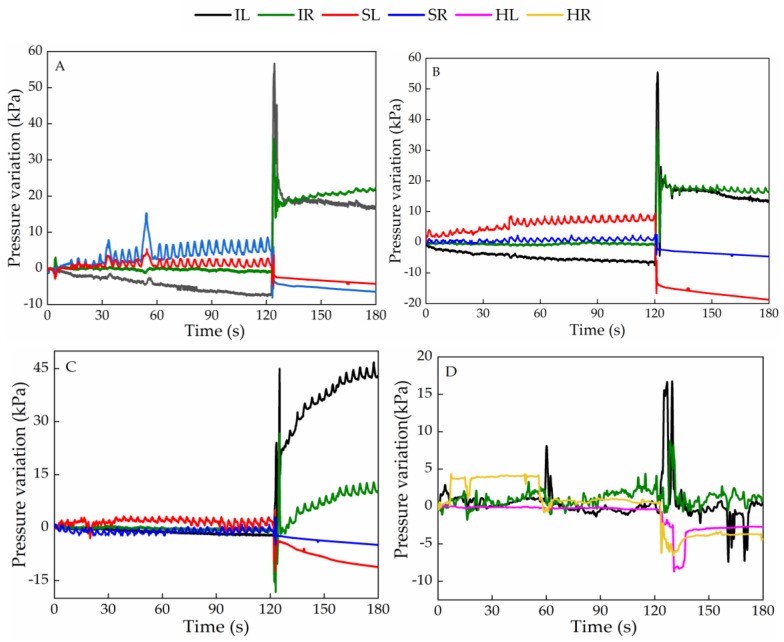

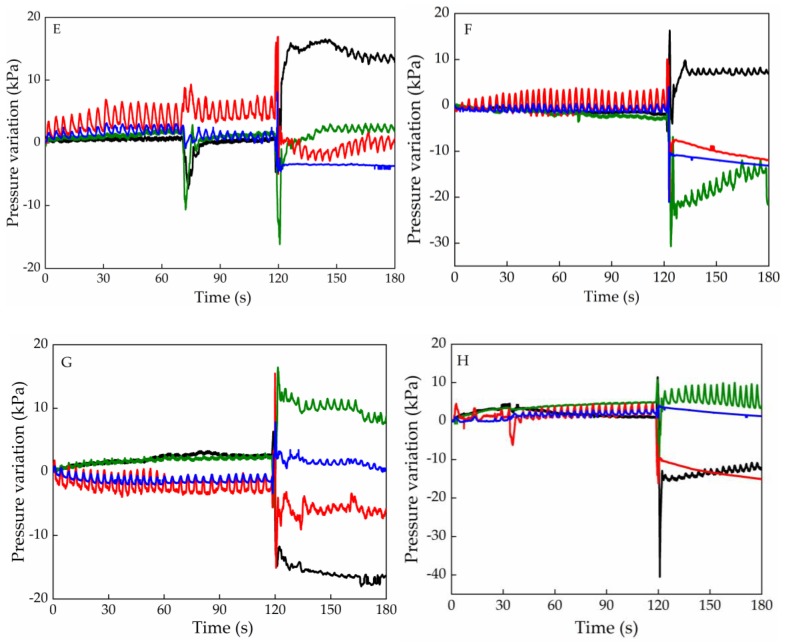
Pressure variation registered in different cells during the pressure relief exercises (**A**–**H**).

Figure 6 shows the response of the sensing cells for the several pressure relief positions. These values were obtained from the Bragg wavelength shift collected during the experimental tests and considering the sensitivity coefficient for each cell (Table 1). The negative pressure values are due to pressure relief in those areas. Upon data analysis, the initial Bragg wavelength was considered at the beginning of the tests, with the subject already seated in the chair, and therefore, with an initial pressure already applied on the sensors. In that way, when the pressure is relieved in a given sensor, there is a decrease in the grating modulation period, and consequently a negative wavelength shift compared with the reference initial Bragg wavelength. For simplicity of nomenclature, each graph is called by the name of the relief position that was tested (A, B, C, D, F, G, and H). However, all the tests started in the NP position, which was maintained during 2 min, followed by the position to be monitored. To simplify the results interpretation, only the signals related to the sensors where significant pressure changes were obtained are represented. Therefore, the response of the cells positioned in the heel is only represented in the graph corresponding to the pressure relief in the feet (D).

In general, all sensors responded to the wheelchair user position changes by sudden variations in the detected signal amplitudes. Also, it should be noted that, the oscillations for the SR, SL, IR, IL signals can be attributed to the subject’s breathing, allowing the estimation of the breathing rate, which is also evaluated in this discussion. In the following, a detailed analysis of the results obtained by the optical sensors (Figure 6) during the various pressure relief positions (Figure 5) is presented.

In test A, the user places herself in a position where the posture angle is approximately 90° and her back is only slightly in contact with the wheelchair. This position change is reflected in the signal detected by the sensors, which in the case of SR there is a reduction of 6.5 kPa, and an increase of 22.1 kPa in the case of IR.

In test B, the user leans forward and places her arms over the legs. As expected, there is a decrease of the pressure registered in the cells positioned in the scapulas, and an increase in the case of the ischiatics, slightly less than in test A, because the upper body is positioned more over the ischiatic sensors in position A than in position B. The higher pressure relief detected in the left scapula intensities (13.7 kPa difference from the right scapula) is an indication that the user was slightly tilted to the left in the NP position, applying a stronger pressure in that sensor, which leads to a higher wavelength shift variation upon the position change (pressure relief).

In test C, the user bends totally forward putting her hands in contact with her feet. In this test, as in the previous ones, the pressure decreases in the scapulas and increases in the ischiatic regions. Again, a greater pressure relief is felt on the left side than on the right side with about 7.2 kPa difference.

In test D, the user is normally seated in the wheelchair but without the footrest, so as expected, a lower pressure is felt in the HL and HR. As the feet are raised, higher pressure is transferred to the ischiatic area, as also detected by the sensors located in that zone. Since this exercise only involves removing of the feet from the wheelchair supports, the pressure exerted by the feet on the insoles, and consequently on the sensing cells is much lower than that exerted on the scapulas and ischiatic regions. Thus, the amplitude of the pressure is different for the pressure values registered for the others pressure relief positions. Further, in previous work [22], a similar cork insole was used for the gait analysis, and higher pressures than those reported in this work were measured, which indicates that there is no limitation on the sensitivity.

In test E, the user places the left arm on a side table 10 cm from the wheelchair so that the right scapula is not in total contact with the wheelchair. In this test a decrease on the signal amplitude of SR and the IR is expected. In this case, a decrease of 3.6 kPa and an increase of 2.2 kPa were obtained, respectively. Contrarily, there is an increase for the case of the sensors at the SL zone (slight initial increase) and IL (increasing 15.0 kPa). As test G corresponds to the same pressure relief exercise, but to the right side, an opposite behavior should be expected. According to the results obtained, there should be greater contact (increasing of the pressure) between the right shoulder blade and the chair, as well as an increase in the pressure felt at the IR. This was detected by the sensors placed at those locations (1.7 and 9.8 kPa for the SR and IR, respectively). As for the IL and the SL, a pressure relief was also detected by the sensors placed in those specific locations with a decrease in the pressure values, 16.4 and 6.5 kPa, respectively.

In test F, the user places her left arm on the side table 20 cm away. In this position both scapulas are no longer in contact with the wheelchair. As expected, a decrease in the pressure on the SL and SR sensors was obtained, 11.7 and 13.1 kPa, respectively. A relief on the IR (14.1 kPa) and an increase in the IL (7.3 kPa) was registered as the user shifts her weight to the left side. In test H, the user repeats the pressure relief position, but this time to the right side, and also in this test the sensors respond as expected: decrease in the pressure felt at the IL (11.9 kPa) and at the SL (15.1 kPa) and slight increase of pressure felt at the IR (6.4 kPa) and SR (1.3 kPa).

Considering the pressure range used in the sensor characterization tests and the pressure amplitude in the graphs of Figure 6, we predict that the proposed architecture could be used by heavier subjects without breaking risk. The pressure values are dependent on the subject´s weight. Regarding the time of contact for the ulcer occurrence, in the literature, there is no agreement about that value, nevertheless, it is known that it varies according to the person’s physiology and skin condition, and the authors from [14] advise the execution of pressure relief exercises during 30 s every 30 min.

**Figure 7:**
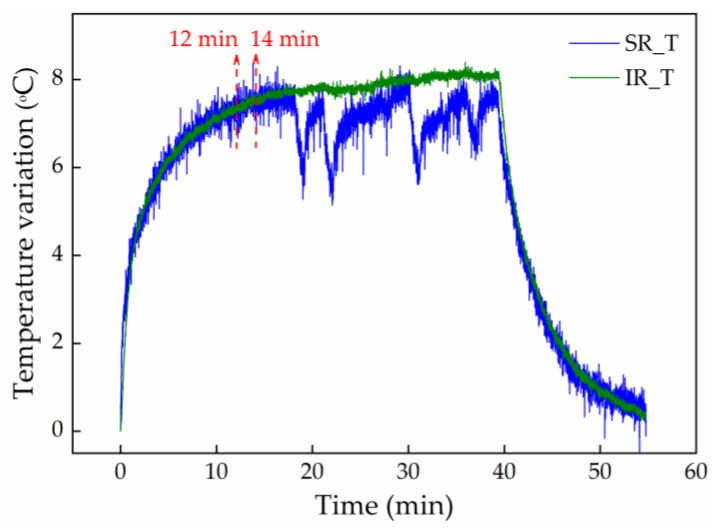
Thermal variation obtained during pressure relief exercises for the sensors positioned in the right scapula and ischiatic.

Figure 7 shows the temperature variation obtained during the pressure relief tests, for the sensors positioned on the right scapula and right ischiatic, identified as SR_T and IR_T, respectively. Note that this experiment was carried out after the wheelchair test. An accentuated increase in the temperature, mainly in the first 10 min (about 7 °C) was observed. Hence, initially, a stabilization period of 14 min was considered, during which the temperature changes as the result of the temperature difference between the environment and the wheelchair user’s body. After the first 14 min of the experiment, and as expected, a small temperature variation (about 0.6 °C) was registered by the sensor positioned in the IR, since the sensor is in contact with the wheelchair user during the whole experimental test (different pressure relief positions). In the case of the sensor positioned in the SR, a notable decrease of the temperature was obtained during the pressure relief exercises involving the loss of contact between the wheelchair user and the sensors. Examples of this situation are the positions B, C, and F. The maximum temperature difference obtained for these cases was about 2 °C. Considering the thermal sensitivity difference between the temperature sensors and the cells (as result of the resin where the FBG was embedded), the 2 °C may lead to an error of around 2 kPa in the pressure determination. However, we point out that this will be the maximum error, and for most of the pressure relief positions it will be lower. In future work, a rigorous temperature control will be carried out, with the inclusion of temperature sensors in the experimental setup. Moreover, each sensing cell could have embedded two FBGs with distinct sensitivities (separation of effects by the matrix method), or, for instance, to be composed of two FBGs, with one of them encapsulated, eliminating the sensitivity to strain/pressure variations.

**Figure 8:**
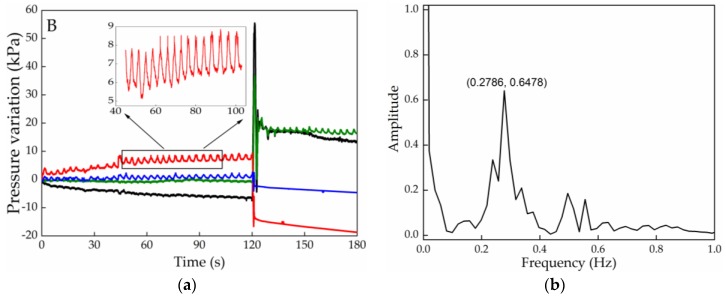
(**a**) Pressure variation registered in different cells, highlighting the SL cell results, during the pressure relief exercise B; (**b**) Fourier transform applied to the optical signal detected by the SL and highlighted in Figure 8a, for wheelchair user breathing rate determination.

Parallel to the pressure detected according to the user’s position, a smaller oscillation in the signal is also detected in the first 2 min in the NP, related to the subject’s breathing rate, Due to the sensing cell positions, this phenomenon is not as clear in the IR and IL zones as it is in the case of the SR and SL, so the breathing rate is estimated from the data obtained for the sensors located at the scapula, Figure 8a.

Figure 8b depicts data of the Fourier transform applied to the initial values registered by the SL cell during the initial part of test B. The highest intensity peak corresponds to a frequency of 0.28 Hz, which is within the frequency range expected for a resting adult (up to 20 breaths/min, which equals a frequency of 0.33 Hz [26]). For the IR position a similar value was also obtained (0.29 Hz). Such results give also a new prospective on the application of this sensing architecture for detecting breathing related pathologies, such as asthma episodes and the moment when they are triggered, or even some psychological related disorders such as anxiety conditions.

## 5. Conclusions

In this work, a reliable solution for monitoring, in real time, the pressure in different zones of a wheelchair was proposed. The system consists of a mesh of six FBGs positioned in prominent bone areas, namely scapulas (R and L), ischiatic zone (R and L), and heels (R and L). The results showed that the use of this system based on optical fiber sensors offers a solution that is reliable, fast, small, and compact, making it an alternative solution to conventional electronic sensors.

The application of this system as an e-Health tool can offer advantages to patients prone to develop neuropathic ulcers in risk zones, where a continuous evaluation of the pressure points can be accessed, and the data retrieved can be continuously transferred to care centers or to associated medical staff. On envisaging a full stand-alone application associated with such architecture, alerts for pressure relief exercises can be given to the user and emergency warnings can be sent to hospitals in critical situations. Additionally, with the proposed system it is possible to retrieve information regarding the breathing rate of the user, important information when considering the pathologies that can be inferred from it, particularly for elderly or debilitated users.

On contemplating the application of this sensing architecture in real context, and considering scenarios of the movement of patients with reduced mobility, special attention should be given to the robustness of the whole sensing system. This includes the improvement of the junction between the epoxy resin cylinder and the outgoing fiber (specially designed protection sleeves can be considered). The protection of the optical fiber connecting the multiplexed sensors should also be optimized.

In the future, it is also planned to compensate the temperature effect during pressure monitoring, and to provide the system with a mobile application that alerts the user to change his/her position to avoid ulcers. Also, the interrogation system will be replaced by including edge filtering techniques for the sensors analysis, which will be a financially affordable solution for the broader use of the proposed technology.

## Figures and Tables

**Table 1:** Sensitivity coefficient of each cell to the applied pressure.

Cell	Sensitivity Coefficient (pm/kPa)
Right heel (HR)	9.6 ± 0.1
Left heel (HL)	10.2 ± 0.2
Right scapula (SR)	18.2 ± 0.2
Left scapula (SL)	17.8 ± 0.3
Right ischiatic (IR)	17.9 ± 0.3
Left ischiatic (IL)	18.5 ± 0.2
